# Secretion of pro‐angiogenic extracellular vesicles during hypoxia is dependent on the autophagy‐related protein GABARAPL1

**DOI:** 10.1002/jev2.12166

**Published:** 2021-12-02

**Authors:** Tom G. Keulers, Sten F. Libregts, Joel E.J. Beaumont, Kim G. Savelkouls, Johan Bussink, Hans Duimel, Ludwig Dubois, Marijke I. Zonneveld, Carmen López‐Iglesias, Karel Bezstarosti, Jeroen A. Demmers, Marc Vooijs, Marca Wauben, Kasper M.A. Rouschop

**Affiliations:** ^1^ Department of Radiation Oncology Radiation Oncology (Maastro) / GROW – School for Oncology and Developmental Biology Maastricht University Medical Centre + Maastricht Netherlands; ^2^ Department of Biomolecular Health Sciences Faculty of Veterinary Medicine Utrecht University Utrecht Netherlands; ^3^ Department of Radiation Oncology Radboud University Medical Center Nijmegen Netherlands; ^4^ Microscopy CORE Lab Maastricht Multimodal Molecular Imaging Institute FHML Division of Nanoscopy University of Maastricht Maastricht Netherlands; ^5^ The M‐Lab Department of Precision Medicine GROW ‐ School of Oncology Maastricht University Maastricht Netherlands; ^6^ Proteomics Center Erasmus University Medical Center Rotterdam Netherlands

**Keywords:** autophagy, exosomes, extracellular vesicles, GABARAPL1, hypoxia

## Abstract

Tumour hypoxia is a hallmark of solid tumours and contributes to tumour progression, metastasis development and therapy resistance. In response to hypoxia, tumour cells secrete pro‐angiogenic factors to induce blood vessel formation and restore oxygen supply to hypoxic regions. Extracellular vesicles (EVs) are emerging as mediators of intercellular communication in the tumour microenvironment. Here we demonstrate that increased expression of the LC3/GABARAP protein family member GABARAPL1, is required for endosomal maturation, sorting of cargo to endosomes and the secretion of EVs. Silencing GABARAPL1 results in a block in the early endosomal pathway and impaired secretion of EVs with pro‐angiogenic properties. Tumour xenografts of doxycycline inducible GABARAPL1 knockdown cells display impaired vascularisation that results in decreased tumour growth, elevated tumour necrosis and increased therapy efficacy. Moreover, our data show that GABARAPL1 is expressed on the EV surface and targeting GABARAPL1^+^EVs with GABARAPL1 targeting antibodies results in blockade of pro‐angiogenic effects in vitro. In summary, we reveal that GABARAPL1 is required for EV cargo loading and secretion. GABARAPL1^+^EVs are detectable and targetable and are therefore interesting to pursue as a therapeutic target.

## 1 INTRODUCTION

The tumour microenvironment is characterised by extreme heterogeneity in oxygenation that primarily arises due to a poorly developed vascular network. Gradients of oxygenation in the tumour vary from normal (5%) values around the vessel wall to complete anoxia in perinecrotic regions. Intertumoural differences in tumour hypoxia greatly influence outcome after therapy, as high levels of tumour hypoxia correlate with therapy resistance and a more aggressive phenotype (Brizel et al., 1997; Harris, 2002; Hockel et al., 1996; Nordsmark et al., 2005). Large differences in tumour hypoxia are observed between patients with otherwise similar clinical characteristics, these are likely to be the result of the cellular ability to increase hypoxia tolerance and to resolve the hypoxic status. To this extent, cells modulate metabolism, angiogenesis, the unfolded protein response (UPR) and autophagy (Rouschop et al., 2010; Wouters & Koritzinsky, 2008). The secretion of angiogenic factors (e.g., Vascular endothelial growth factor [VEGF], Platelet derived growth factor [PDGF], and angiogenin) into local tissues and stroma is essential in triggering angiogenesis. These factors stimulate the adjacent quiescent vasculature to grow new vessels into the tumour and resolve hypoxia (Hanahan & Folkman, 1996). Associations between autophagy‐related proteins and secretory events have been described and contribute to tumour progression (Keulers et al., 2016).

In recent years, exosomes and microvesicles (collectively referred to as extracellular vesicles [EVs]) have emerged as mediators of intercellular communication. EVs are small (70–1000 nm) membrane vesicles that are secreted by cells to shuttle complex biological information such as lipids, mRNA's, miRNA's, soluble, and transmembrane proteins. EVs are highly enriched in tetraspanins (CD81, CD63, and CD9), that are widely used as markers for EV identification (Andreu & Yáñez‐Mó, 2014; Théry et al., 2018; Van Deun et al., 2017). Data accumulated over the years indicates that tumour‐derived EVs are essential in enhancing tumour growth through stimulation of proliferation, influencing the immune system by triggering immunosuppressive responses (Belting & Christianson, 2015; Ekström et al., 2014; Hong et al., 2019; Ludwig et al., 2017; Mincheva‐Nilsson & Baranov, 2014) and they are potent inducers of angiogenesis in vitro and in vivo (Ko et al., 2019; Kucharzewska et al., 2013; Lindmo & Stenmark, 2006; Ludwig et al., 2018; Zonneveld et al., 2019). The role of hypoxia in EV secretion is largely unknown, although some studies show increased EV production and changes in cargo sorting with pro‐tumoural effects in these conditions (Kumar & Deep, 2020; Zonneveld et al., 2019).

Exosomes are derived from the endosomal compartment of the cell. The endosomal system comprises membranous structures involved in endocytic, degradative and secretory pathways and is linked to the autophagy machinery (Xu et al., 2018). Internalised cargo is rapidly targeted to early endosomes, where it is recycled back to the plasma membrane or processed to late endosomes. Early endosomes mature into late endosomes/multivesicular bodies (MVB) through the sorting of new cargo derived from the Golgi *en route*. During this process, referred to as endosomal maturation, intraluminal vesicles (ILVs) are formed by inward budding of the limiting membrane of the endosome (Huotari & Helenius, 2011). MVB content can be degraded through lysosomal fusion or secretion after fusion with plasma membranes. After fusion, ILV are secreted as exosomes. Recent data indicates that the autophagy machinery is involved in EV secretion and cargo loading (Guo et al., 2017; Leidal et al., 2020; Sanwald et al., 2020).

Members of the Gamma‐aminobutyric acid receptor‐associated protein (GABARAP) protein family are associated with the lysosomal degradation pathway, autophagy and intracellular receptor trafficking. They share structural characteristics, but have unique biological functions (Schaaf et al., 2016). Previously, we showed that the expression of GABA type A receptor associated protein like 1 (GABARAPL1), a member of the GABARAP protein family, is induced during hypoxia due to activation of the protein kinase R (PKR)‐like endoplasmic reticulum kinase (PERK) arm of the UPR (Keulers et al., 2015). In this report, we describe the discovery of a unique subset of EVs that are specifically secreted by hypoxic cells and are dependent on hypoxia‐induced GABARAPL1 expression. Importantly, we observed that GABARAPL1 is expressed on the surface of these EVs after secretion. We exploited this therapeutic entry point by using blocking antibodies against GABARAPL1, which resulted in inhibition of EV function in vitro. Furthermore, we show that tumour growth and regrowth after irradiation is delayed after silencing GABARAPL1 in tumour xenografts, as a result of reduced vascularisation and enhanced tumour necrosis. In conclusion, we show a unique role for GABARAPL1 in tumour progression through secretion of EVs by hypoxic cells.

## 2 MATERIAL AND METHODS

### 2.1 Cell culture

HT29 (colorectal adenocarcinoma), U87 (glioblastoma), and MCF7 (breast cancer) and immortalised human umbilical cord endothelial cell lines (EC‐RF‐24, Applied Biological Materials, Canada) were maintained in 10% FCS (Sigma) supplemented DMEM and RPMI (Lonza), respectively. For EV isolations, 30% FCS was depleted of EVs by ultracentrifugation overnight (SW32 rotor, BeckmanCoulter, 100.000 RCF). The origins of the cell lines were authenticated by STR analysis (Identicell, Denmark). Hypoxia exposure experiments were performed using a modular atmosphere controlled system (Don Whitley Scientific). Cells were exposed to hypoxia for 16–20 h over night. Doxycycline‐inducible (1 μg/ml) shRNA targeting the 3′UTR of GABARAPL1 [5′TTACCTTACTTCATACTTGCCC 3′] or control shRNA (scrambled) [5′CGAGGGCGACTTAACCTTAGG 3′] was achieved through lentiviral pTripZ (Thermo Scientific) expression. Independent doxycycline inducible hairpins targeting the GABARAPL1 coding sequence were expressed using pLKO.1 based lentiviral expression (5′ACAGTGATGAGAGTGTCTATG 3′). GABARAPL1 knockdown was induced 3 days prior to experiments. Reintroduction of the wild type GABARAPL1 coding sequence was achieved through expression in pQCXIH backbones using lentiviral transduction. The pBABE‐puro mCherry‐EGFP‐LC3B was a gift from Jayanta Debnath (Addgene plasmid # 22418). Expression was achieved through retroviral transduction.

### 2.2 Sample processing for quantitative mass‐spectrometry

After 24 h ambient oxygen or hypoxia exposure, conditioned culture medium (serum free) was collected and spun down for 10 min 300×g. Subsequently, secreted proteins were concentrated using centrifugal filters (Amicon Ultra, 3K). Protein concentration was determined using Bradford assay (Bio Rad). 18 mg of secreted proteins were sonicated in 12 mM Sodiumdeoxycholate (SDC),12 mM sodium laurylsarcosinate (SLS) in 100 mM Tris/HCl (pH 9.0) (Method modified from Masuda et al., 2009). Disulfide bonds were reduced in 5 mM dithiothreitol at 50°C for 30 min and free sulfhydryl groups were then alkylated in 10 mM iodoacetamide for 30 min at room temperature in the dark. The protein mixture was diluted five times with 50 mM ammonium bicarbonate and digested with trypsin (1:50 w/w) at 37°C overnight. Digestions were acidified with trifluoroacetic acid (TFA) to a concentration of 0.5% to precipitate the SDC and SLS. SDC and SLS were removed by centrifugation.

Desalting and reductive dimethylation of free amino groups was performed on C18 stagetips (Method modified from Wilson‐Grady et al Volume 61, Issue 3, 15 june 2013, Pages 277‐286). Briefly, tryptic peptides from secreted proteins were separately loaded onto stagetips and washed with 200 μl 0.1% TFA and subsequently with 200 μl 0.25 M MES (2‐(N‐morpholino) ethanesulfonic acid) pH 5.5. Samples 1L: MCF7 SCR N, 3L: MCF7 GABARAPL1 A, 4L: HT29 SCR A and 6L:HT29 GABARAPL1 A were labelled by loading 200 μl light labelling solution (0.4% formaldehyde [Sigma] and 60 mM sodium cyanoborohydride [Sigma] in 0.25 M MES [pH 5.5] onto the stagetip). Samples 2H: MCF7 Control hypoxia and 5H: HT29 control hypoxia were labelled by loading 200 μl heavy labelling solution (0.4% ^13^C‐D2 formaldehyde [Sigma Isotec] and 60 mM (D3)‐sodium cyanoborodeuteride [Sigma Isotec] in 0.25 M MES [pH 5.5]). The stagetips were washed with 200 μl 0.1% formic acid and labelled peptides were eluted with 100 μl 50% acetonitrile with 0.1% Formic acid. Next eluates from samples 1L+2H, 2H+3L, 4L+5H and 5H+6L were mixed and dried by vacuum centrifugation.

### 2.3 Mass spectrometry analysis

All LC–MS/MS analyses were performed on a Q Exactive mass spectrometer (Thermo Scientific, San Jose, CA). Dried mixed light and heavy labelled samples were resuspended in 0.5% formic Acid in 3% acetonitrile and loaded onto an Easy spray column (Pepmap RSLC, C18, 2 μm, 75 μm × 25 cm Thermo Fisher Scientific). Peptides were separated using the Thermo Fisher Scientific Easy Nano LC1000 (buffer A = 0.1% formic acid and buffer B = 0.1% formic acid in acetonitrile) using a gradient of 0%–28% buffer B for 90 min, with a flow rate of 300 nl/min. Each Data collection cycle in the Q Exactive consisted of one full MS scan (300–1750 m/z) followed by 15 data dependent MS/MS scans.

### 2.4 Bioinformatics analysis

Raw data files were processed using MaxQuant software suite (version 1.5.2.8, Tyanova et al., 2016) for identification and relative quantification of proteins. A false discovery rate (FDR) of 0.01 for proteins and peptides and a minimum peptide length of six amino acids were required. The Andromeda search engine was used to search the MS/MS spectra against the Human Uniprot database. A maximum of two missed cleavages were allowed. Q Exactive spectra were analysed using MaxQuant's default settings for Orbitrap spectra, including a main search peptide and MS/MS match tolerance of 4.5 and 20 ppm, respectively. The maximum precursor ion charge state used for searching was seven, and the enzyme specificity was set to trypsin. Further modifications were cysteine carbamidomethylation (fixed) as well as protein methionine oxidation. For dimethylation labelled samples 28,031 KDa on lysine and the peptide N‐terminus (light) and 36,076 KDa on lysine and the peptide N‐terminus (heavy) were added to the search parameters. The minimum number of razor and unique peptides was set to 1. Heavy‐to‐light (H/L) ratios were calculated using MaxQuant's default settings. Uniprot human_2013_06 database was used for bioinformatics analysis. Gene enrichment analysis was done using the gene ontology website (http://geneontology.org/)

### 2.5 EV purification and labelling

EVs were isolated and purified based on the protocol described previously (Thery et al., 2006). In short, cells were cultured in growth medium that was depleted from FCS‐derived EVs by overnight ultracentrifugation at 100.000*g*. After exposure of 5E^6^ HT29 or U87 cells to hypoxia or ambient oxygen, EVs were collected from the culture supernatant by differential ultracentrifugation. Samples were depleted from cellular debris and apoptotic bodies by centrifuging for 10 min 200*g* at RT, followed by 20 min 2000*g* and 30 min at 16.000*g* at 4°C. Finally, EVs were pelleted (60 min 100.000 RCF, SW41 rotor [Beckman Coulter]) labelled with PKH67 (Sigma Aldrich) as described previously (Van Der Vlist et al., 2012) and subsequently floated to their buoyant density into a linear sucrose gradient (0.4–2.0 M sucrose) by ultracentrifugation for 16 h (200.000 RCF, SW41 rotor [Beckman Coulter]). Twelve sucrose fractions of 1 ml were then collected from bottom to top and either diluted with PBS for subsequent high‐resolution flow cytometric analysis or immunoblotting. EVs were labelled with anti‐GABARAPL1 (1:50, protein tech group) and alexa594 (1:50, goat anti‐rabbit IgG, Invitrogen) and PKH67 for general EV staining. Labelled EVs were floated into a linear sucrose gradient as described above. For ultracentrifugation SW32 and SW41 rotors (Beckman Coulter) were used. We have submitted all relevant data from our experiments to the EV‐TRACK knowledgebase (Van Deun et al., 2017) (EV‐TRACK ID: EV200121) (EV‐Metric score 61%).

### 2.6 High‐resolution flow cytometry

Flow cytometry analysis of individual EVs was performed on a BD Influx flow cytometer (Becton Dickinson, Brussels, Belgium) that was adapted and optimised for the analysis of single submicron‐sized‐particles. The detailed configuration of this platform is fully described by Van Der Vlist et al. (2012). For detection of EVs, the system was triggered on the fluorescence derived from PKH67‐labelled EVs passing the first laser. The fluorescence threshold level was determined by measuring 0.22 μm‐filtered PBS, allowing an event rate of ∼10 events/s. To allow comparisons between experiments, yellow‐green fluorescent 100‐nm and 200‐nm polystyrene beads (FluoSpheres; Invitrogen) were used as reference in every experiment. Samples were measured at event rates lower than 10,000 events/s to prevent swarm detection (Kormelink et al., 2016).

### 2.7 Tunable resistive pulse sensing

Size distribution was measured using TRPS (qNano gold, Izon) with Izon Control Suite 3.2 Software and SKP200 calibration beads (Izon, Chirstchurch, New Zealand) using NP200 nanopores. The current was adjusted to 125 nA. Where possible, at least 500 EV counts were acquired per sample. Samples used for TRPS were freshly prepared by differential ultracentrifugation followed by sucrose gradient purification. Samples were diluted with solution Q.

### 2.8 EV immunocapture

20E6 cells were cultured in EV depleted medium and exposed to severe hypoxia (O_2 _<0.02%) for 16 h. EVs were isolated by differential ultracentrifugation. The 100K pellet was resuspended in PBS and incubated with GABARAPL1 antibody (11010‐1‐1P, Protein tech group) and IgG isotype control (30000‐0‐AP, Protein tech group) coupled protein A beads (Dynabeads, ThermoFisher) overnight. Precipitated EVs were washed with PBS and lysed in RIPA buffer. Proteins were visualised by immuno blot analysis.

### 2.9 Matrigel plug assay

EVs from 135E6 cells cultured in EV depleted medium were isolated and purified using size exclusion chromatography (SEC). First, medium was collected and depleted of debris and apoptotic bodies using differential ultracentrifugation (10 min 200 RCF, 20 min 2000 RCF, and 30 min 16.000 RCF, respectively). Supernatant was concentrated using centrifugal filter units (Amicon Ultra 15, 100K, Milipore). Concentrated supernatant was loaded onto a Sepharose® CL‐2B column (Telos SPE, Cole Parmer) and 12 fractions were collected. Fractions 7–9 were further concentrated using centrifugal filter units (Microcon®, PL‐10, Millipore) and mixed with matrigel (Corning) (1:10 V/V). EV containing matrigel was “plugged” in the flank of nu/nu mice (*n* = 3). After 7 days plugs were removed and frozen in liquid nitrogen. Vessel growth and infiltrating cells were assessed at three different depths as described in the section Immunohistochemistry and Image Processing.

### 2.10 BrdU proliferation assay

Cells were seeded at a density of 23.500 cells/cm^2^ and allowed to attach overnight. Next, cells were exposed to normoxia (21% O_2_) or moderate hypoxia (0.2% O_2_) for 24 h. Subsequently, BrdU was added to the culture medium (10 μM) for 1 h after which cells were fixed in 4% paraformaldehyde for 20 min. After permeabilization with 0.1% triton X‐100 for 20 min, the cells were treated with 2 M HCl for 30 min at 37°C and twice with 0.1 M borate for 5 min. After blocking in 10% FCS for 30 min, cells were incubated with primary antibody (anti‐BrdU, ABD serotec, OBT0030S) 1/50 for 90 min and with secondary antibody (FITC‐Goat Anti‐Rat IgG, Invitrogen, 62‐9511) 1/200 for 1 h. Nuclei were stained with Hoechst for 10 min. Images were taken using the Nikon Eclipse Ts2 fluorescent microscope. The amount of (BrdU‐positive) nuclei was quantified using Image J software and cell proliferation was expressed as the percentage of BrdU positive nuclei from the total amount of nuclei.

### 2.11 Annexin V/ dead cell assay

Expression of Annexin V was assessed using flow cytometry. Cells were seeded and exposed to normoxic and severe hypoxic conditions (24 h). All cells, including floating cells, were collected and stained with LIVE/ DEAD™ (Invitrogen) staining kit according the manufacturer's manual. Subsequently, Annexin V was stained using Violet Annexin V/ dead cell apoptosis kit (Invitrogen) according the manufacturer's manual. Gating was set on single cells. Apoptotic cells were defined as AnnexinV +/ Aqua – and necrotic cells as AnnexinV+/Aqua+. Cells were measured using a BD FACSCanto II flow cytometer (BD Biosciences) and analysed using Flowjo software (v10.7.2 FlowJo, Ashland, OR, USA).

### 2.12 Immunoblotting

Cells were lysed, separated by SDS‐PAGE and transferred to PVDF membranes. Sucrose gradient fractions were pooled as indicated, diluted with PBS and centrifuged 60 min and 100.000*g* using SW41 rotors (Beckman Coulter). Pellets were resolved in 1× non‐reducing loading buffer. Transferred proteins were probed using antibodies directed against GABARAPL1 1:1000 (11010‐1‐1P, Protein tech group), Actin 1:200.000 (Clone 4, MP Biomedicals), CD81 (clone JS81), CD63 (clone H5C6), and CD9 (clone ML13) 1:1500 (BD Biosciences) and MAP1LC3B 1:1500 (polyclonal, Cell Signaling), visualized using respective HRP linked secondary antibodies 1:5000 (Cell Signaling) and detected using SuperSignal West Pico Chemiluminescent Substrate (Pierce Biotechnology, Thermo Fisher Scientific).

### 2.13 Angiogenesis array

Cells were exposed to ambient oxygen and severe hypoxia (O2 <0.02%). After 24 h, growth medium was collected and centrifuged for 10 min 300*g* to deplete cell debris. Angiogenesis array was performed according to the manufacturer's manual (Proteome Profiler Human Angiogenesis Array Kit, R&D Systems).

### 2.14 Tube formation

Immortalized HUVEC cells were plated in 96‐well plates coated with 50 μl matrigel (Corning) and stimulated with EVs for 16 h. EVs were harvested by the previously described differential ultracentrifugation from the cell culture supernatant of HT29, U87, and MCF7 control, knockdown and control cell‐lines that were exposed for 16 h to severe hypoxia (O2 <0.02%). Blocking antibody and EVs were incubated 30 min before stimulation. EGM2 medium (Lonza) was used as positive control.

### 2.15 High resolution confocal microscopy

After fixation (3,8% paraformaldehyde) cells were permeabilized with 0.05% Tween in PBS and blocked using 5% normal goat serum. Cells were probed with anti‐GABARAPL1 (protein tech group, 11010‐1‐1P, 1:100), MAP1LC3B (polyclonal, cell signalling, 1:100), CD81 (clone JS81), CD63 (clone H5C6), CD9 (clone ML13) (BD bioscience1(1:100)), EEA1 (Abcam), PI3KClass 3 (cell signalling), and hoechst33342 (Sigma Aldrich). Goat anti‐rabbit Alexa‐488 (Invitrogen) and Goat anti‐mouse A647 were used for visualization. Bound antibodies were analysed using Leica TCS SP8 CARS confocal microscope. Images were deconvoluted using Huygens software.

### 2.16 Tumour models

HT29 and U87 cells were transplanted in the flanks of nu/nu mice (1E6 cells/ 50 μl matrigel). Doxycycline was administered through the drinking water (2 g/L doxycycline, 5% sucrose). Tumour volume was measured by calliper measurement in three orthogonal diameters (L × W × H). Animals were injected with pimonidazole (60 mg/kg i.p. hypoxyprobe‐1; Chemicon) and BrdU (30 mg/kg i.p.; Sigma‐Aldrich) 60 min prior to killing. Tumour volumes (mm^3^), corrected for skin thickness (‐0.5 mm), were calculated with the formula ((Width(mm)‐0,5)*(Length(mm)‐0,5)*(Height(mm)‐0,5)*PI)/6. All animal experiments were conducted in accordance with national guidelines and approved by the animal ethics committees of Maastricht University (study number 2011‐138).

### 2.17 Immunohistochemistry and image processing

Frozen, acetone‐fixed sections were stained by using anti‐pimonidazole (Chemicon), 9F1 (rat monoclonal antibody to mouse endothelium; Department of Pathology, Radboud University Nijmegen Medical Centre) as described previously (Rouschop et al., 2010). The slides were scanned by a computerised digital image processing system by using a high‐resolution intensified solid‐state camera on a fluorescence microscope (Axioskop; Zeiss) with a computer‐controlled motorised stepping stage. Tumour necrosis was evaluated, relative to the total tumour area, morphologically by using H&E staining. Tumour hypoxic fraction and vascular density (structures per square millimetre) were determined relative to the viable tumour tissue (necrosis excluded).

### 2.18 Click‐it chemistry

After 16 h exposure to severe hypoxia or ambient oxygen, medium was changed to methionine free for 1 h. Subsequently, L‐AHA (50μM) was added and incubated for 60 min. Medium was changed to normal and collected after 6–8 h. Proteins were precipitated with chloroform/methanol and resuspended in 0.5 M tris‐HCl 1%SDS. Click‐it reaction was done according to the manufacturer's manual (Click‐It® Invitrogen). Proteins were loaded on SDS‐page gel and transferred to PVDF membrane, blocked with 2% casein. Newly synthesised proteins were visualised using streptavidin‐HRP overnight at 4°C in blocking buffer

### 2.19 Cryo‐electron microscopy

U87 derived hypoxic vesicles were isolated by differential ultracentrifugation as described previously and labelled with anti‐GABARAPL1 (1:50). For visualisation GABARAPL1 was labelled with anti‐Rabbit IgG Gold antibody (anti‐GABARAPL1, 11010‐1‐1P, protein tech group) (20 nM). A thin aqueous film of the sample was formed on a Quantifoil R2/2 grid (Quantifoil GMbH, Jena BRD) by applying 2.5 μl of sample and blotting away excess liquid. The grid was held by tweezers in the environmental chamber (20°C and more than 95% relative humidity) of the Vitrobot mark IV (a vitrification robot from FEI) (Iancu et al., 2006). After blotting, the grid with the thin aqueous film was rapidly vitrified by plunging into ethane cooled to its melting point (‐180°C) by liquid nitrogen. The vitrified specimen was transferred to the cold stage (Gatan cryo‐holder) of the transmission electron microscope (FEI‐T12 Biotwin). The pictures were taken with a FEI‐Eagle‐4K‐CCD camera.

### 2.20 BSA‐gold internalisation

After hypoxia exposure (24 h), cells are exposed to BSA‐gold (10 nm) (dept. of cell biology, University Medical Centre, Utrecht) for 10 min. After BSA‐gold internalisation (10 min), cells were fixed with 2.5% glutaraldehyde in phosphate buffer. Cells were kept in the fixative during 24 h at 4°C. Then, they were washed with 0.1 M cacodylate buffer and potsfixed with 1% osmium tetroxide in the same buffer containing 1.5% potassium ferricyanide during 1.5 h at 4°C. Then the samples were embedded in agarose and dehydrated in ethanol, infiltrated with Epon resin during 2 days, embedded in the same resin and polymerised at 60°C during 48 h. Ultrathin sections were obtained using a Leica Ultracut UCT ultramicrotome and mounting on Formvar‐coated copper grids. They were stained with 2% uranyl acetate in water and lead citrate. Then, sections were observed in a Tecnai T12 electron microscope equipped with an Eagle 4kx4k CCD camera (Thermo Fisher Scientific, The Netherlands).

### 2.21 Statistics

Data were analysed with GraphPad Prism software. Multiple testing was done using a repeated‐measures ANOVA with a Bonferroni post‐hoc test. Student's *t* test was used for single comparisons Statistical test used are described in each figure legend. All animal data are presented as mean ± SEM. Data were considered statistically significant when *p* was less than 0.05.

## 3 RESULTS

Gradients of oxygenation in the tumour may vary from normal (5%) values around the vessel wall to complete anoxia in perinecrotic regions. Previously we showed that increased GABARAPL1 expression during hypoxia is regulated by the PERK arm of the ER‐stress regulated unfolded proteins response (UPR) (Keulers et al., 2015), a mechanism typically activated by cells exposed to severe hypoxia.

**FIGURE 1:**
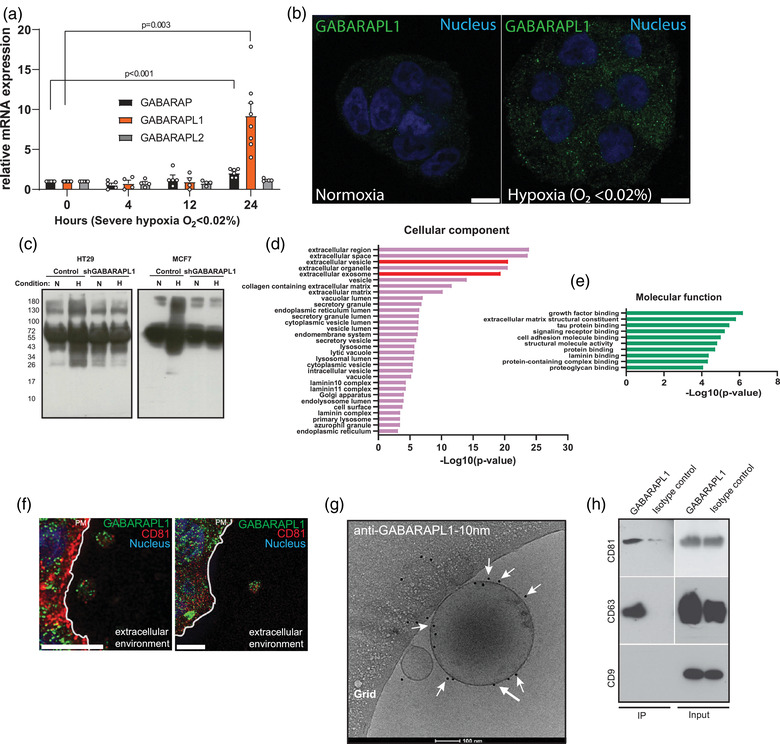
GABARAPL1 is secreted on extracellular vesicles. (a) mRNA expression levels of the GABARAP family during hypoxia (test unpaired, 2‐tailed, mean ± SEM *n* > 4 independent experiments). (b) Confocal micrographs of endogenous GABARAPL1 (green) during normoxia and severe hypoxia (<0.02 O_2,_ 16 h). Nucleus is depicted in blue. Scalebar = 10 μm. (c) A representative image of immunoblots showing a decrease of secreted L‐AHA incorporated protein by GABARAPL1 knockdown HT29 and MCF7 cells. (d+e) Gene enrichment analysis of differentially secreted proteins by HT29 GABARAPL1 knockdown cells. (f) Confocal micrograph of GABARAPL1 (green) and CD81 (red) indicate colocalization on vesicular extracellular structures. Nucleus is depicted in blue. The plasma membrane is indicated by a white line. Scalebar = 5 μm. (g) Cryo‐electron micrograph of EVs after immuno gold‐labelling with anti‐GABARAPL1 indicates that GABARAPL1 expression is retained on the EV membrane after secretion. Size‐bar is 100 μm. Some GABARAPL1‐10 nm gold beads are indicated by white arrows. (h) Immunoblot of immune‐ captured EV show that the tetraspanins reside on GABARAPL1^+^ EV

GABARAPL1 is a member of the GABARAP protein family consisting of GABARAP, GABARAPL1 and GABARAPL2. Interestingly, GABARAPL1 mRNA expression is most responsive to severe hypoxia (O_2 _<0.02%) in HT29 cells (Figure 1a). Immunolabelling of endogenous GABARAPL1 confirms upregulation during hypoxia and reveals a focal pattern in cells (Figure 1b). Nevertheless, GABARAPL1 expression is not exclusively limited to hypoxia, but is also induced by other stresses such as mitochondrial stress (Figure S1A), suggesting that GABARAPL1 is used by cells in response to or to alleviate cellular stresses.

To further evaluate the distribution and localisation of GABARAPL1 during hypoxia, we fluorescently tagged GABARAPL1 (mCherry‐GABARAPL1) and monitored its distribution by live cell imaging. These experiments revealed that mCherry‐GABARAPL1 is trafficked towards the cells’ periphery (Video S1). The localisation and specific movements of these GABARAPL1+ vesicles motivated us to investigate if GABARAPL1 is involved in secretory events.

To determine if GABARAPL1 is involved in secretion, we analysed the total secreted protein content in the culture medium of GABARAPL1 knockdown cells during ambient oxygen (21% O_2_) and severe hypoxic (O_2 _<0.02%) conditions. Click‐it chemistry using incorporation of the methionine homolog L‐azidohomoalaine (L‐AHA) allowed us to specifically label nascent cell culture‐derived secreted protein. Secreted proteins were then isolated by precipitation and subsequently biotin labelled by click‐it chemistry and visualised by streptavidin‐HRP immunoblotting. We observed that during hypoxia protein secretion is increased in control cells. Silencing GABARAPL1 resulted in a decrease of secreted protein in MCF7 and HT29 cells in these conditions (Figure 1c).

To identify which proteins are dependent on GABARAPL1 for secretion, we identified secreted proteins through SILAC‐based mass spectrometry. After isolation from the culture medium, total protein of control and GABARAPL1 knockdown cells were labelled with heavy and light isotopes respectively, pooled and analysed by mass spectrometry (for a table listing all differentially expressed proteins, see Table S1). Functional enrichment analysis revealed that proteins that are dependent on GABARAPL1 expression for secretion are associated with EVs such as exosomes (Figure 1d) and involved in growth factor signalling (Figure 1e), suggesting that GABARAPL1 is involved in EV secretion.

These findings prompted us to investigate if GABARAPL1 colocalises with markers of EVs during hypoxia. Immunolabelling revealed that GABARAPL1 is located in the extracellular environment on structures that are positive for the tetraspanin CD81, a widely used EV marker (Figure 1f). To confirm GABARAPL1 secretion on EVs, EVs from the culture medium of cells exposed to severe hypoxia were isolated. Immunogold‐labelling of intact, unfixed EVs followed by cryo‐EM visualization revealed that GABARAPL1 is, at least, present at the surface of secreted EVs (Figure 1g). Intriguingly, although GABARAPL1 expression can be detected on small and larger vesicles, not all EVs express GABARAPL1 (data not shown), suggesting that GABARAPL1 marks a subset of EVs. Characterisation of GABARAPL1^+^ EVs by immunocapture reveals that the tetraspanins CD81 and CD63 are present on GABARAPL1^+^ EVs. CD9 expression appears to be less abundant and remained below the detection limit (Figure 1h).

### 3.1 GABARAPL1 is required for endosomal maturation

**FIGURE 2:**
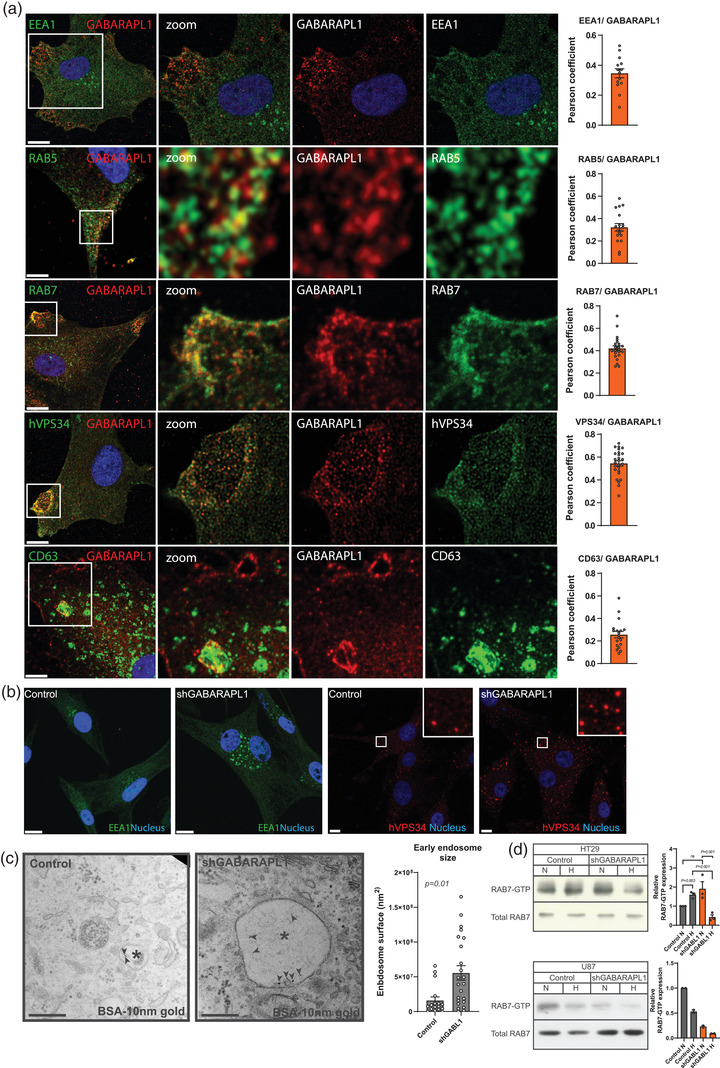
GABARAPL1 is required for endosomal maturation. (a) confocal micrographs of U87 cells exposed to hypoxia show localization of GABARAPL1 (red) to markers of early (EEA1 and RAB5, red) and late endosomes (RAB7 and CD63, green) and hVPS34 (green) during hypoxia (O_2 _<0.02, 16 h). Right panels represent quantifications (>20 cells) from independent experiments. Shown is mean ±SEM. Nucleus is depicted in blue (Scalebar = 10 μm). (b) Confocal micrographs of hypoxic U87 GABARAPL1 knockdown cells labelled for EEA1 and hVPS34 show increased size of endosomal compartments. (c) Electron micrograph shows increased size of early endosomes after BSA‐10 nm gold internalization. Endosomes are indicated by asterix, BSA‐gold particles are indicated by arrows. Right panel shows quantification of >20 BSA‐10 nm gold positive structures (*p* = 0.02 *t*‐test unpaired, 2‐tailed, mean ± SEM). (d) Left panels: Westernblot analysis of RAB7 activity assay shows decreased RAB7 activity (RAB7‐GTP) in HT29 and U87 GABARAPL1 knockdown cells. Right panels: western blot quantifications of independend experiments (*n* = 3, *t*‐test unpaired, 2‐tailed, mean ± SEM, U87 is done *n* = 2).

EVs, more specifically exosomes, are formed by inward budding of the endosomal membrane during maturation from early to late endosomes. Endosomal maturation is characterised by replacement of RAB5 on the early endosome by RAB7, a highly controlled process referred to as RAB conversion (Rink et al., 2005). If GABARAPL1 is functionally involved in EV cargo sorting or release, it is expected to overlap with endosomal compartments such as early and late endosomes. To determine where GABARAPL1 is located in the endocytic pathway, we assessed colocalisation of GABARAPL1 with markers of early endosomes (RAB5, EEA1), late endosomes/MVB (RAB7, CD63) and early/late endosomes (hVPS34/ PI3KC3) of cells exposed to severe hypoxia (16–18 h). These studies reveal that GABARAPL1 is located in the early and late endosome phase of the pathway (Figure 2a). In particular colocalisation with RAB7 and VPS34 and late endosome/MVB and lysosomal marker CD63‐positive structures was observed. Interestingly, colocalisation of GABARAPL1 with CD63 positive structures was not observed for all CD63 positive structures, suggesting distinct fate, endosomal stage or GABARAPL1‐independent processing.

Based on these findings, we hypothesized that GABARAPL1 has a functional role in endosomal processing. To test this hypothesis, we silenced GABARAPL1 through inducible shRNA expression and assessed the endosomal markers by confocal immunofluorescence microscopy. Both the early endosomal marker EEA1 and hVPS34 showed increased number and enlarged endosomal structures (Figure 2b). These findings were confirmed by electron microscopy by internalization experiments. BSA‐gold particles localize to early endosomes after 10 minutes (Raiborg et al., 2002; Schmid, 1988). Quantification after hypoxia exposure (O_2 _<0.02%) revealed a 3‐fold increase (1.80E5–5.8E5, respectively) in early endosomal size in GABARAPL1 deficient cells (Figure 2c). This indicates a blockade in processing at the early endosomal stage.

Active, GTP‐bound RAB7 (RAB7‐GTP), is essential for endosomal maturation and motility and required for downstream processing of the late endosome (Huotari & Helenius, 2011; Stein et al., 2003). Therefore, RAB7 activity by pull down analysis of RAB7‐GTP and total RAB7 was assessed. In line with previous data, GABARAPL1 deficient cells display decreased RAB7‐GTP in both U87 and HT29 cells (Figure 2d). Taken together these data indicate that GABARAPL1 overlaps with endosomal compartments, particularly in the late endosome stage, and silencing GABARAPL1 causes enlarged early endosomes, suggesting that GABARAPL1 is critical for endosomal maturation.

### 3.2 GABARAPL1 is not essential for (macro‐)autophagy

**FIGURE 3:**
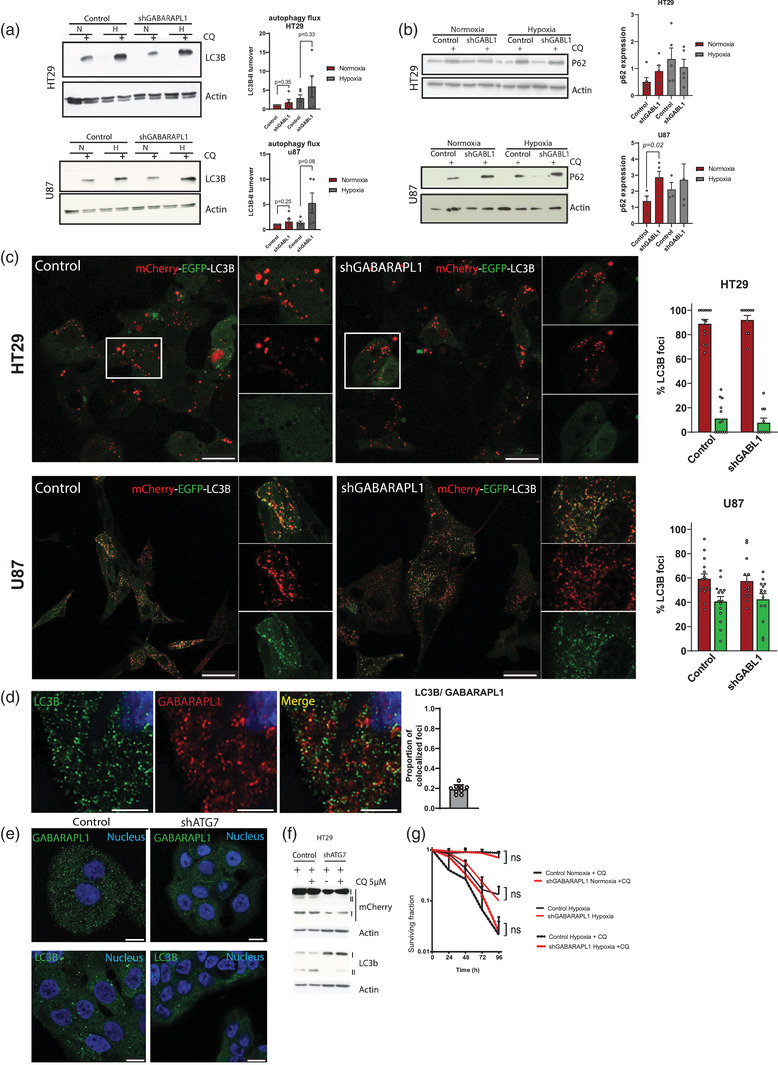
GABARAPL1 is not essential for (macro)‐ autophagy. (a) Representative western blots show no change in autophagic flux after silencing GABARAPL1 in HT29 and U87 cells after 16 h hypoxia exposure (N = normoxia, H = Severe hypoxia). Right panels show quantified LC3B‐II flux/ turnover (LC3B‐II+CQ – LC3B‐II) *n* = 6, *t*‐test unpaired, 2‐tailed, mean ± SEM. (b) Western blots of P62/SQSTM‐1 turnover in GABARAPL1 deficient cells. Shown are representative westernblot of at least three independent experiments. Right panels show quantifications of western blots (*t*‐test unpaired, 2‐tailed, mean ± SEM). (c) Confocal micrographs of mCherry‐eGFP‐LC3B expressing cells. Right panels show quantifications of >20 cells from independent experiments. Shown is mean ± SEM. Scalebar = 25 μm. (d) Expression of fluorescently labelled endogenous GABARAPL1 (red) and LC3B (green) show partial co‐localization in HT29 cells. Nucleus is depicted in blue. Quantification of co‐localized LC3B and GABARAPL1 foci. Scalebar = 5 μm. (e) Confocal micrographs showing that GABARAPL1 and LC3B are targeted to intracellular vesicles in an ATG7 dependent manner. GABARAPL1 and LC3B are depicted in green, nucleus in blue. Scalebar = 10 μm. (f) Western blots of mCherry‐GABARAPL1 show that GABARAPL1 is conjugated to PE is ATG7 dependent. (g) GABARAPL1 is not required for survival and is still sensitive to chloroquine during severe hypoxia (<0.02% oxygen), as assessed in HT29 cells by clonogenic survival assays (*t*‐test unpaired, 2‐tailed, mean ± SEM *n* = 3 independent experiments)

The endosomal pathway is closely related to autophagy. It has been suggested that GABARAPL1 plays a role in the later stages of autophagosome formation (Weidberg et al., 2010) and acts as an autophagy receptor for selective autophagy (Schaaf et al., 2016). Additionally, hVPS34 and RAB7 are shown to have a functional role in autophagy. VPS34 is normally found in protein complexes: VPS34 complex I is dedicated to autophagy while VPS34 complex II is involved in endocytic trafficking and cargo sorting (Lindmo & Stenmark, 2006; Vanhaesebroeck et al., 2010). Despite several studies that show promising links between GABARAPL1 and autophagy associated processes, its exact functional role has, to the best of our knowledge, not been established so far. Therefore, we examined the functional role of GABARAPL1 in (macro)‐autophagy. The gold standard used to determine the level of autophagy is to determine the level of LC3B turnover (the autophagic flux) (Klionsky et al., 2016). This is the net difference of LC3B‐II levels in conditions with and without an autophagy inhibitor, such as chloroquine. We observed that silencing GABARAPL1 does not significantly affect the autophagic flux (Figure 3a). In addition, turnover of the autophagy receptor p62/ sequestosome‐1 (SQSTM1) was measured as a secondary measure of flux. In line with LC3B‐II turnover, silencing GABARAPL1 did not affect p62/ SQSTM1 turnover during severe hypoxic conditions. During ambient oxygen exposure, GABARAPL1 knockdown increased p62/ SQSTM turnover in U87 cells. Additionally, we employed the mCherry‐eGFP‐LC3B as another indicator of autophagy activity. This reporter system enables measurement of autophagic activity through quenching of the eGFP in the late/ acidic stages of autophagy. No differences in autophagy activity were observed (Figure 3c).

In line with these observations, co‐expression of fluorescently labelled fusion proteins revealed that GABARAPL1 foci only partially colocalise with LC3B in cells (Figure 3d). Nevertheless, similar to LC3B, GABARAPL1 recruitment to vesicles is dependent on the autophagy machinery as indicated by reduced GABARAPL1 foci in ATG7 deficient cells (Figure 3e). To further evaluate whether GABARAPL1 is modified in an ATG7 dependent manner, we overexpressed mCherry‐tagged GABARAPL1. Comparable to LC3B, GABARAPL1 is modified in an ATG7 dependent manner as indicated by the lack of GABARAPL1‐II in ATG7 deficient cells (Figure 3f).

During cell stress, such as hypoxia, autophagy is activated to support survival. We, and others, have shown before that pharmacological or genetic inhibition of autophagy decreases cell survival during these conditions. Therefore, if GABARAPL1 would be required for the execution of autophagy, one would expect a survival disadvantage of GABARAPL1 knockdown cells compared to controls. However, survival studies with GABARAPL1 knockdown cells show no decrease in survival (Figure 3g). Additionally, incubation of GABARAPL1 knockdown cells with the autophagy inhibitor chloroquine, still sensitises these cells to hypoxia. To exclude that this is mediated by autophagy independent effects of chloroquine, survival of ATG7 deficient cells was assessed. ATG7 deficiency results in sensitisation to severe hypoxia, but no additional effects of chloroquine are observed (Figure S3). These results indicate that the observed effects on GABARAPL1 deficient cells (Figure 3g) are mainly due to the inhibition of autophagy.

Together, these results indicate GABARAPL1 is not essential for (macro‐)autophagy, but is at least partially, dependent on proteins that are also used for the execution of autophagy. These data however, do not exclude a functional role of GABARAPL1 in selective forms of autophagy.

### 3.3 GABARAPL1 is required for EV cargo loading and secretion

ILV are formed during endosomal maturation by inward budding of the endosomal membrane. ILV containing endosomes, referred to as MVB, release ILV into the extracellular environment as exosomes. Our mass spectrometry identified decreased secretion of EV associated proteins (CD81, HSP70) by GABARAPL1‐deficient cells, we therefore questioned if GABARAPL1 is required for EV secretion.

**FIGURE 4:**
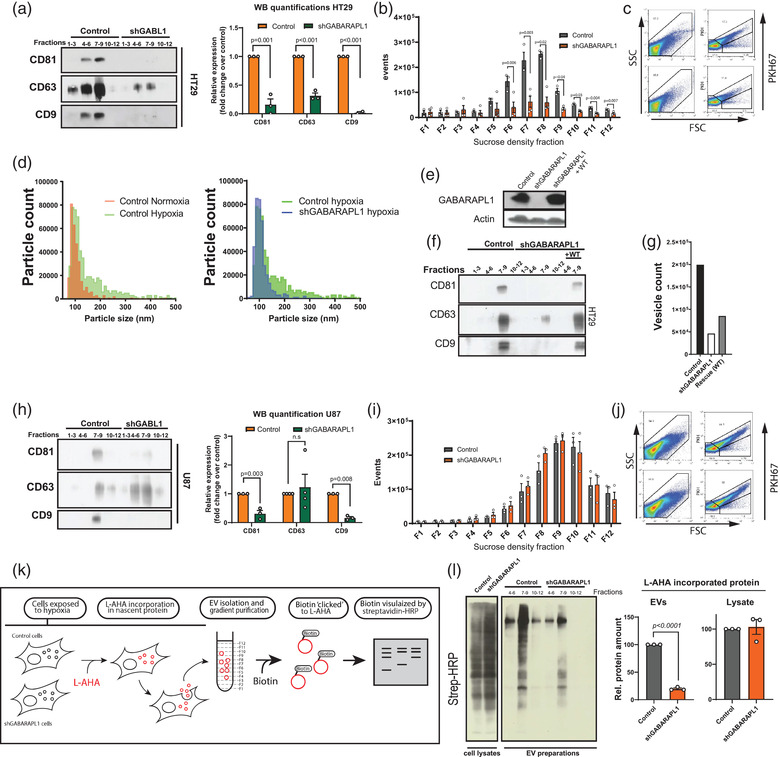
GABARAPL1 is required for EV secretion and cargo sorting. (a) Secreted EV marker evaluation after EV isolation by differential ultracentrifugation followed by immunoblot analysis reveals reduced marker abundance in secreted EVs of HT29 cells. Sucrose density fractions are pooled per 3. blots are from three independent experiments (*t*‐test unpaired, 2‐tailed, mean ± SEM). Right panels show quantifications of western blots from four independent experiments (*n* = 4, mean ± SEM). (b) High resolution flow cytometry of sucrose fractions after centrifugation indicates reduced EV secretion in HT29 GABARAPL1 deficient cells exposed to hypoxia (*n* = 4 ± SEM). (c) Corresponding scatterplots of high‐resolution flow cytometry of fraction 7. (d) Size distribution analysis of secreted EVs by resistive pulse sensing shows that a larger subpopulation is secreted during hypoxia (green) (left panel) which is GABARAPL1 dependent (Blue, right panel). (e) Re‐expression of wild‐type (WT) GABARAPL1 restores the marker composition of secreted EVs as assessed by (f) immunoblot analysis (representative example of *n* = 4) or (g) high resolution flow cytometry after sucrose density purification. Plotted are total counts of fraction 4–12. Shown is 1 representative example of *n* = 2 independent experiments. (h) Secreted EV marker evaluation after EV isolation by differential ultracentrifugation followed by immunoblot of secreted EVs derived from U87 cells show changed marker composition upon GABARAPAL1 silencing. Sucrose density fractions are pooled per 3. Western blots are from at least three independent experiments. Right panel shows quantifications of three experiments (*t*‐test unpaired, 2‐tailed, mean ± SEM). (i) High resolution flow cytometry of sucrose fractions show no difference in EV counts secreted by GABARAPL1 deficient U87 cells (O_2 _<0.02%). (j) Corresponding scatterplots of high‐resolution flow cytometry of fraction 7. (k) Graphical presentation of analysis of L‐AHA incorporated protein and subsequent loading into EV. (l) Left panel: Western blot analysis show a decrease of L‐AHA incorporated protein loaded in EV in GABARAPL1 knockdown cells. Right panel: quantification of three independent experiments (*n* = 3, *t*‐test unpaired, 2‐tailed, mean ± SEM)

To test this, we analysed the marker composition and quantity of EVs released by HT29 and U87 GABARAPL1 deficient cells after expose to severe hypoxia. GABARAPL1 deficiency results in reduced secretion of the EV‐associated proteins CD81, CD63, and CD9 with two independent shRNAs (Figures 4a and S4A). Absolute differences in EV secretion was quantified by high‐resolution flow cytometry. High‐resolution flow cytometry was shown to be a sophisticated method to detect single labelled EVs (Van Der Vlist et al., 2012) and is considered as a true single EV quantification method. Using this method, we observed that EV secretion during hypoxic conditions is dramatically decreased after GABARAPL1 silencing in HT29 cells (Figure 4b,c). Due to the small size and low refractive index of EVs, high‐resolution flow cytometry is limited in determining EV size. To determine the accurate size distribution of the secreted EVs, we used Tunable Resistive Pulse Sensing (qNano). We observed that during hypoxia, HT29 cells secrete a larger sized (>150 nm) subpopulation (Figure 4d, left panel) which is completely dependent on GABARAPL1 expression for secretion (Figure 4d, right panel).

To exclude that the observed effects are not the result of off target effects from the shRNA, GABARAPL1‐expression was rescued with expression of the coding sequence (WT), not targetable by the hairpin (Figure 4e). This resulted in normalisation of EV‐marker secretion in HT29 cells (Figure 4f) and increased secretion of EV (Figure 4g), indicating that the hairpin is GABARAPL1 specific. Additionally, GABARAPL1 can be modified through C‐terminal lipidation with phosphatidylethanolamine (PE) to allow membrane association (Nath et al., 2014; Sou et al., 2006). In line, we observed that ATG7 is required for GABARAPL1 modification (Figure 3f) and that intracellular vesicular recruitment of GABARAPL1 is dependent on ATG7 expression (Figure 3e). To evaluate GABARAPL1 lipidation on EV phenotype determination, EV secretion in ATG7 deficient cells was determined. Similar to GABARAPL1 knockdown, ATG7 deficiency resulted in decreased EV secretion and a decrease in the EV markers CD81, CD63 and CD9 (Figure S4B). These data suggest that lipidation of GABARAPL1 is important for EV composition. ATG7 may be involved in EV secretion in a complementary manner.

In comparison to HT29 cells, U87 show a slightly different secretory phenotype. Although similar effects in CD81 and CD9 secretion was observed, CD63 appeared non‐affected. Also, EV counts were not decreased in U87 cells after knockdown with two independent shRNAs (Figures 4h and S4C), suggesting that U87 cells have an additional GABARAPL1 independent sorting mechanism. Intrigued by these observations, we further investigated this finding. Interestingly, HT29 and U87 show different degrees of colocalisation of intracellular GABARAPL1 and CD63. In U87 cells (Figure 2a) CD63 positive patches were observed that are devoid of GABARAPL1. In contrast, in HT29 (Figure S2), all CD63‐positive patches colocalize with GABARAPL1. These data suggest a reliance on distinct mechanisms of EV secretion.

### 3.4 GABARAPL1 is required for cargo loading

Endosomal maturation occurs due to cargo being sorted into the endosome “*en route*.” Since our findings indicate that GABARAPL1 is required for endosomal maturation, we hypothesised that cargo sorted into EVs might also be GABARAPL1 dependent. To test this, we tracked newly synthesised protein sorted into secreted EVs using click‐it chemistry (Figure 4k). Although total protein synthesis is comparable between GABARAPL1 pro‐ and deficient cells, a decrease in L‐AHA incorporated protein in EVs isolated from GABARAPL1 deficient cells is observed (Figure 4l).

### 3.5 GABARAPL1 is required for hypoxic cells to stimulate angiogenesis

**FIGURE 5:**
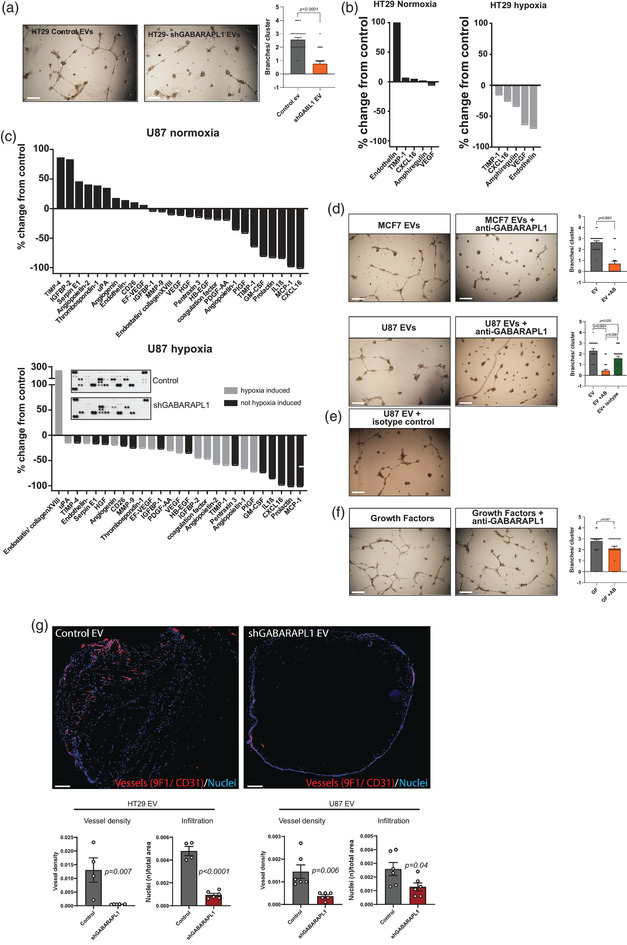
GABARAPL1 deficient cells display decreased angiogenic potential. (a) Left Panels: Hypoxic HT29 derived EVs induce tube formation in HUVEC cells after stimulation with EVs isolated from control but not GABARAPL1 deficient cells. Right panels: quantifications of branches per cluster of HUVEC cells (*n* > 25 clusters, Mann‐Whitney, 2‐tailed mean ± SEM) Scalebar is 200 μm. (b) Quantification of antibody‐arrays of U87 conditioned medium of control and GABARAPL1 knockdown cells. Plotted is the fold change from control. Insert is a representative example of antibody array membranes. (c) Quantification of antibody‐array of HT29 control and shGABARAPL1 cells. (d) Tube formation is blocked by GABARAPL1 antibodies. HUVEC were stimulated with hypoxic EVs from U87 and MCF7 wild‐type cells. Tube‐formation is blocked when EVs are incubated with GABARAPL1 antibodies. Right panels show quantifications of branches per cluster of HUVEC cells (*n* > 25 clusters, Mann‐Whitney, 2‐tailed mean ± SEM) Scalebar is 200 μm. (e) Isotype control show increased tube formation compared to GABARAPL1 targeting antibodies (*n* > 25 clusters, Mann‐Whitney, 2‐tailed mean ± SEM) Scalebar is 200 μm. (f) Soluble growth factors with antibodies (VEGF, FGF2, IGF, EGF) induce tube formation. Right panels show quantifications of branches per cluster of HUVEC cells (*n* > 25 clusters, Mann‐Whitney, 2‐tailed mean ± SEM) Scalebar is 200 μm. (g) Matrigel plug assays show decreased CD31+ vessel formation when stimulated with EVs from GABARAPL1 deficient cells. Shown are cross sections of matrigel plugs. Vessels are stained with 9F1/CD31 antibodies and depicted in red. Nuclei of infiltrated cells are stained with DAPI and depicted in blue. For quantifications, cross sections from three different depths from three independent animals/plugs were used (*t*‐test unpaired, 2‐tailed, mean ± SEM three animals/plugs per group) scalebar is 1 mm

To alleviate tumour hypoxia, cells release factors that stimulate angiogenesis (Harris, 2002). Although the most studied angiogenic factors were not identified, mass spectrometry analysis identified factors, dependent on GABARAPL1 expression, that could change the balance of a pro *versus* anti angiogenic secretome, such as Stromal derived factor 4 (SDF4), Lysyl oxidase‐like 4 (LOXL4), Growth differentiation factor 15 (GDF15), SerpinE2, Jagged1, Trefoil Factor 1 (TFF1) (Table S1) (Chi et al., 2021; Dong et al., 2018; Li et al., 2019; Pedrosa et al., 2015; Sasahira et al., 2021; Shi et al., 2019). HUVECs exposed to EVs derived from hypoxic cells have previously been found to stimulate endothelial tube formation in vitro (Kucharzewska et al., 2013; Tadokoro et al., 2013). To test whether the changes in EV secretion and angiogenic factor secretion after GABARAPL1 depletion altered the angiogenic potential of hypoxic cells, endothelial tube formation assays were performed after incubation with EVs derived from hypoxic control and GABARAPL1 knockdown cells. A clear reduction in angiogenic potential was observed (Figure 5a).

To determine which factors are involved in GABARAPL1 mediated angiogenesis, the levels of 55 angiogenesis associated proteins were determined in cell culture supernatant using antibody arrays (Figure 5b). Quantification of factors above the detection limit reveals that secreted levels of several factors are altered in GABARAPL1 knock down HT29 cells (Figure 5b), U87 (Figure 5c), and MCF7 cells (Figure S5A). These effects are most pronounced during hypoxia. Many of the angiogenic factors produced during hypoxia are dependent on transcriptional activity of hypoxia inducible factors (HIF) (Harris, 2002). Analysis of transcriptional targets of HIF signalling (VEGF, carbonic anhydrase 9, glucose transporter 1) indicated that HIF signalling is not dependent on GABARAPL1 (data not shown). Additionally, GABARAPL1 expression is HIF1 independent (Keulers et al., 2015). Combined these results indicate that the observed effects are independent of HIF signalling and suggest that GABARAPL1 dependent sorting (Figure 4l) is required for effective release.

GABARAPL1 expression at the surface of secreted EVs (Figure 1g) allows targeting. To evaluate if EV membrane‐expressed GABARAPL1 is required for vesicle mediated effects, GABARAPL1 was targeted with antibodies. HUVEC stimulation with EVs in the presence of GABARAPL1 targeting antibodies results in abrogation of endothelial tube formation (Figure 5d,e). Endothelial tube formation by direct addition of soluble growth factors (VEGF, FGF2, IGF, and EGF) could not be prevented by GABARAPL1‐targeting antibodies (Figure 5f), confirming the EV specific effect of GABARAPL1 targeting.

To further evaluate the angiogenic properties of GABARAPL1^+^ EV, we performed in vivo matrigel plug assays with EV containing plugs. In line with the in vitro analyses, plugs containing EVs from GABARAPL1 deficient cells displayed decreased CD31^+^ vessel growth (Figure 5g), confirming the direct effect of GABARAPL1^+^EV on angiogenesis. This was associated with decreased infiltration of cells, suggesting that, besides changes in pro‐angiogenic properties of GABARAPL1^+^EV, some chemoattractants are also dependent on GABARAPL1 for sorting into EVs (Figure 5g, right panels).

Taken together these data show that GABARAPL1^+^ EV have direct effects on angiogenesis. This is mediated, at least in part, through changed loading of pro‐angiogenic factors.

### 3.6 GABARAPL1 supports tumour progression through enhancement of angiogenesis

**FIGURE 6:**
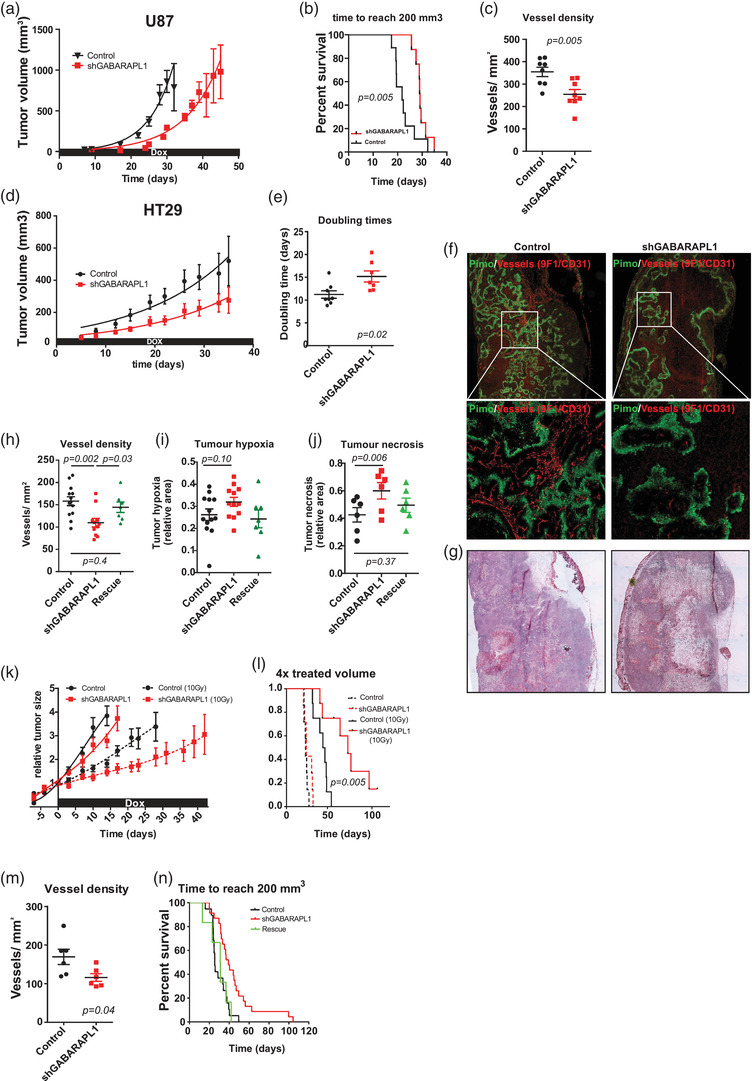
GABARAPL1 contributes to tumour progression by enhancing angiogenesis. (a) Growth curve of U87 xenografts: GABARAPL1 knockdown (*n* = 8) and control xenografts (*n* = 9) (Mean ± SEM). (b) Survival curves of U87 xenografts reaching 200 mm^3^ (Wilcoxon log rank), *p* = 0.05). (c) GABARAPL1 knockdown is associated with decreased vessel density (*p* = 0.005, *t*‐test unpaired, 2‐tailed, mean ± SEM). (d) Growth curve of knockdown (*n* = 8) and control (*n* = 8) HT29 xenografts. (e) Doubling times of HT29 xenografts (*p* = 0.02 *t*‐test unpaired, 2‐tailed, mean ± SEM). (f) IHC analysis of hypoxia (pimonidazole, green) and vessels (9F1, red) of HT29 xenograft cross sections) (g) with matching H&E. (h) Vessel density (control *n* = 13, shGABARAPL1 *n* = 11, rescue *n* = 7), *t*‐test unpaired, 2‐tailed, mean ± SEM). (I) Hypoxia quantification of control (black, *n* = 13), GABARAPL1 knockdown (red, *n* = 12), and rescue tumours (*n* = 7, green) (*t*‐test unpaired, 2‐tailed, mean ± SEM). (j) Tumour necrosis, control (*n* = 6 (black) and GABARAPL1 knockdown (*n* = 6, red) (control VS knockdown, *p* = 0.006) and rescue tumours (green) after contralateral implantation (control VS rescue *p* = 0.37, *t*‐test unpaired, 2‐tailed, mean ± SEM). (k) Normal growth (continuous line) and tumour growth after single dose of 10 Gy (dotted lines) of control and GABARAPL1 knockdown tumours (*n* = 8 per group). (L) Kaplan‐Meier curve of tumours reaching 4x treated volume Control 10 Gy (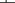) and shGABARAPL1 10 Gy (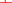); (Gehan‐Breslow‐Willcoxon test, *p* = 0.005). (M) Vessel density after irradiation (control *n* = 6, shGABARAPL1 *n* = 6, *p* = 0.04 *t*‐test unpaired, 2‐tailed, mean ± SEM). (N) Kaplan‐Meier curves of tumours reaching 200 mm^3^. Control (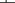, *n* = 19), GABARAPL1 knockdown (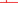, n = 23), and Rescue shGABARAPL1 (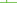, *n* = 6) Gehan‐Breslow‐Willcoxon test. Control VS Rescue *p* = 0.4, shGABARAPL1 VS Rescue *p* = 0.006)

Our data indicate that GABARAPL1 is required for the secretion of hypoxia‐induced EVs that are required to elicit pro‐angiogenic effects. To test this in a more complex setting, U87 cells were subcutaneously grown in the flanks of female nu/nu NMRI mice. Cells were engineered to express shRNA under a doxycycline‐inducible promoter, which was administered through the drinking water. GABARAPL1 deficiency resulted in delayed tumour growth (Figure 6a, b) and was associated with reduced vessel density (31% difference, *p* = 0.005 (Figure 6c), as assessed by 9F1 immunohistochemistry. Despite lower vessel density no differences in tumour hypoxia, as assessed by pimonidazole immunohistochemistry, or differences in vessel perfusion and vessel size were observed (Figure S4A, B).

Comparable results were obtained with HT29 xenografts. We observed that GABARAPL1 knockdown reduced tumour growth (Figure 4d) as illustrated by an increase in tumour doubling times from 11 to 15 days (36%) (Figure 6e). Pimonidazole and 9F1 immunohistochemical analysis (Figure 6f) revealed that in this model the vessel density was also reduced after GABARAPL1 knockdown (Figure 6h, f). The levels of hypoxia in the viable tissue did not differ from controls (Figure 6i). Interestingly, morphology assessment using H&E staining indicated that GABARAPL1 deficient tumours displayed increased tumour necrosis (Figure 6g and j). Blood vessel size (Figure S6B) and perfusion remained unchanged (Figure S6F). Also, assessment of cellular characteristics did not reveal any differences in morphology (Figure S6C), apoptotic markers (annexin V) or proliferative capacity (BrdU incorporation) (Figure S6D, E) that would provide alternative explanations for our observations. These results further support the conclusion that the observed delayed tumour growth of GABARAPL1 knockdown xenografts is due to reduced vascular development.

Next, we questioned if GABARAPL1 targeting would yield beneficial effects in a more clinically relevant situation (e.g., targeting an existing tumour). The doxycycline inducible promoter allows establishment of tumours with intact GABARAPL1 expression, which can be transiently blocked by addition of doxycycline to the drinking water. No significant difference in growth rates between, as wild‐type established, control and GABARAPL1 knockdown tumours was observed (Figure 6k, l, dotted lines) indicating no impact on existing vasculature. Next, we questioned if GABARAPL1 targeting could potentially be used as an adjuvant therapy. For this, we allowed xenografts to reach a volume of 150 mm^3^, irradiated the tumours with a single, tumour specific dose of 10 Gy followed by GABARAPL1 knockdown through doxycycline administration. We observed that targeting GABARAPL1 after irradiation significantly reduced regrowth of the tumours (Figure 6k, i) which was again associated with reduced vessel density (Figure 6m). This finding is potentially interesting for incurable tumours treated with palliative intent. Although GABARAPL1 knockdown was induced after irradiation, irradiation‐induced cell death may take several days. To exclude effects on intrinsic radio sensitivity after GABARAPL1 depletion, clonogenic survival was determined after irradiation. No effect of GABARAPL1 knockdown was observed on intrinsic radiosensitivity of cells (Figure S6I), suggesting that the observed growth delay is caused by reduced tumour‐supportive angiogenesis. Interestingly GABARAPL1 expression in downregulated 24 h after irradiation in vitro (Figure S6J).

Finally, the effects of GABARAPL1 deficiency on tumour growth and TME might be caused by cell intrinsic changes, rather than by EVs. For example, we and others have demonstrated that GABARAPL1 is involved in anterograde trafficking of several receptors, including EGFR, which might influence tumour cell proliferation (Keulers et al., 2015). To determine to what extent the observed effects are EV‐mediated or are a consequence of cell intrinsic changes, animals were implanted with two control (control), two GABARAPL1 knockdown (shGABARAPL1), or one GABARAPL1 knockdown combined with one control tumour on the contralateral flank (rescue). Again, GABARAPL1 deficiency results in delayed tumour growth compared to control tumours (Figure 6n). Importantly, the delayed tumour growth was partially rescued if the same animal was implanted with a control tumour as well as a GABARAPL1 deficient tumour (Figure 6n). In addition, the reduced vessel density (Figure 6h) and increased tumour necrosis (Figure 6j) of GABARAPL1‐deficient tumours was also resolved. These results support our observation that GABARAPL1 is important for the development and secretion of hypoxia‐induced EVs that are required to induce angiogenesis. These results indicate systemic delivery of GABARAPL1^+^ EVs that mediate distant communication.

## 4 DISCUSSION

GABARAPL1 is a member of the GABARAP protein family, a group of proteins contributing to cellular homeostasis by mediating essential cellular processes such as autophagy and the trafficking of receptors to the plasma membrane (Schaaf et al., 2016). As a member of the LC3/GABARAP protein family, GABARAPL1 is presumed to fulfil a role in the general execution of autophagy. We unexpectedly observed that GABARAPL1, is not essential for the general execution of (macro) autophagy. In line, recent studies show that GABARAPL1 is probably more involved in selective forms of autophagy (Li et al., 2020; Yamamoto et al., 2021). However, functional redundancy of GABARAP proteins in the execution of autophagy have been described (Nguyen et al., 2016) and potentially explains why silencing GABARAPL1 only has negligible effects on autophagic activity.

In this study we describe that GABARAPL1 is required for the secretion of EVs and growth factors during hypoxia and thereby contributes to tumour growth in various tumour cell types. Furthermore, we show that GABARAPL1, and to a lesser extend GABARAP, but not GABARAPL2, expression is induced during severe hypoxia, emphasising the unique functions of the distinct GABARAP family members. In addition to hypoxia, other stresses also induce the expression of GABARAPL1, suggesting that GABRAPL1 is used by cells as a general stress response mechanism, or as a mechanism to alleviate stress. Although we only used severe hypoxia in our in vitro experimental setup, this does not preclude that other stressors or external factors are able to induce comparable effects.

EVs, more specifically exosomes, are formed during endosomal maturation by inward budding of the limiting endosomal membrane. The maturation of early to late endosomes is a result of cargo, derived from internalised vesicles and trans‐golgi, sorted into the endosome *en route*. Although the exact mechanism is still unknown, we observed that GABARAPL1 is required for the sorting of cargo into the endosome leading to endosomal maturation and, inherently, the sorting of cargo into EVs. Our data reveals that silencing GABARAPL1 resulted in enlarged early endosomes and decreased RAB7 activity suggesting a functional impairment of the endosomal pathway, probably due to a decrease in cargo sorted into the endosome. In line, GABARAPL1 deficient cells sorted less protein cargo into an equal number of secreted EVs, potentially by inhibited endosome maturation. Hyperactivated RAB5 can lead to defective endosomal maturation and sorting, resulting in increased early endosomal size as a result of increased fusion (Bucci et al., 1992; Kaur & Lakkaraju, 2018; Stenmark et al., 1995; Wegener et al., 2010). RAB‐GTPase activity is turned on or off by guanine exchange factors (GEFs) or GTPase‐activating proteins (GAPs), respectively. TBC1D containing proteins are shown to have GAP capacity. We (unpublished data) and others (Popovic et al., 2012) have found that TBC1D is a GABARAPL1 binding partner, supporting a potential link between GABARAPL1 and endosomal maturation through regulating RAB activity.

Striking differences were observed between the cell lines used regarding levels of secreted EVs. GABARAPL1 deficient HT29 cells show a dramatic decrease of secreted EVs. In contrast, silencing GABARAPL1 in U87 cells only resulted in a change of EV composition. More specifically, EV markers CD9 and CD81 were decreased, but CD63 expression was remarkably stable in secreted EVs. Interestingly, U87 cells seem to have GABARAPL1 negative, but CD63 positive cytoplasmic structures. This suggests that U87, compared to HT29, have an additional sorting and secretory mechanism, independent of GABARAPL1. In contrast to the tetraspanins CD81 and CD9, CD63 mainly localises to late endosomes and lysosomes (Pols & Klumperman, 2009). Incorporation of cargo into ILV is largely dependent on the ESCRT machinery. However, sorting of CD63 is independent of the ESCRT machinery, but is mainly dependent on ceramide (Trajkovic et al., 2008). This could mean that GABARAPL1 dependent sorting works in close collaboration with the ESCRT machinery.

Our studies indicate a role for GABARAPL1 in the biogenesis and protein loading of EVs. Previously, Leidal et al. have shown that its homologue, LC3B, is required for sorting of RNA cargo to, and secretion of, EVs (Leidal et al., 2020), suggesting that the LC3 family members each have unique functions in EV biogenesis. Interestingly, another homologue, GABARAP, is secreted in EVs. GABARAP EV proximity labelling revealed overrepresentation of RNA binding proteins and mitochondrion associated proteins (Sanwald et al., 2020) and further supports a role for LC3/GABARAP family proteins in specific cargo sorting.

In line with our results, Leidal et al. showed that GABARAPL1 is sorted into EVs in an ATG7 dependent manner and that ATG7 deficiency results in decreased EV secretion (Leidal et al., 2020). These results indicate that the biogenesis of EV and autophagosomes is, at least in part, orchestrated by the same mechanism. In support, other ATG proteins are associated with EV biogenesis and secretion too. For example, formation of an ATG12‐ATG3 complex allows association with the ESCRT protein ALIX (Murrow et al., 2015). Disruption of the ATG12‐ALIX complex results in hampered EV biogenesis and secretion (Leidal et al., 2020; Murrow et al., 2015). The formation of autophagosomes depends on complexation of ATG12 with ATG5 and ATG16L. Besides ATG12, deficiency in either ATG5 or ATG16L1 also results in decreased EV production through controlling LC3B meditated late endosomal V_1_V_0_‐ATPase activity (Guo et al., 2017).

Combined, these results indicate that autophagy (related proteins) and the biogenesis and processing of EVs are dependent on the same machinery. How the decision of secretion *versus* degradation is made, remains to be elucidated.

Functional assays show that hypoxia‐induced EVs enhance tube formation in HUVEC cells, an effect that can be abrogated by silencing or targeting GABARAPL1. In line with these results, we observed that in two xenografted tumour types, GABARAPL1 deficient tumours display decreased vessel density. Surprisingly, in contrast to HT29 xenografts, GABARAPL1 deficient U87 tumours did not grow slower compared to control tumours, despite a reduced vessel density. These effects may be related to the already very high vascular density of glioblastoma tumours and the difference in cargo sorting mechanisms.

Furthermore, as we showed previously, GABARAPL1 is also required for hypoxia induced membrane expression of EGFR and other hypoxia associated factors (Keulers et al., 2015). Thus, inhibition of GABARAPL1 could potentially alter tumour growth by affecting intrinsic factors, for example, downstream signalling of EGFR, leading to decreased cell proliferation, resulting in decreased tumour growth. Although in vitro experiments suggest that the observed effects on tumour development are GABARAPL1^+^ EV related, it is challenging to discriminate if the observed effects in tumour growth in vivo are solely due to GABARAPL1^+^ EV or also caused by cell intrinsic changes. To test this, GABARAPL1 knockdown tumours and their direct environment should be targeted by GABARAPL1+ EVs. Direct administration of isolated GABARAPL1^+^ EVs in vivo has several disadvantages such as EV dosage, as well as biodistribution and bioavailability. Furthermore, EV isolation might change the in vivo behaviour. To overcome these issues, control tumours were implanted at the contralateral flank in the same animal. Zomer et al. showed that distant tumours release and exchange functional biomolecules, mediated by EVs (Zomer et al., 2015). Indeed, we found that the decreased growth of GABARAPL1 knockdown tumours could be rescued partially by circulating factors secreted by control tumours in a GABARAPL1 dependent manner.

Since we found that hypoxic cell‐derived GABARAPL1^+^ EVs contribute to tumour growth and GABARAPL1 is present on the outer membrane of secreted EVs, GABARAPL1 might also be a new therapeutic target. As a proof of principle, we showed that the angiogenic function of GABARAPL1^+^ EVs can be blocked in vitro by the use of antibodies directed against GABARAPL1. This was supported by our observation in vivo that inhibition of GABARAPL1 following irradiation contributed to delayed tumour regrowth. Extrapolated to cancer patients this could mean that GABARAPL1 blocking antibodies could inhibit angiogenesis or neovascularisation and thereby inhibit tumour growth and increase susceptibility to therapeutic interventions.

In conclusion, we show for the first time that GABARAPL1 is important for tumour development, through secretion of a specific subset of pro‐angiogenic EVs, marked by GABARAPL1 expression. Our data show that these EVs are detectable, targetable and are interesting to pursue as a therapeutic target. 

## AUTHOR CONTRIBUTIONS

T.G.K. developed the hypothesis, designed the experimental approach, analysed and interpreted the data, performed experiments, wrote the manuscript. S.F.L. Performed High‐Resolution Flow cytometry experiments. K.G.S performed western blots, DNA‐cloning, processed samples, analysed data and performed fluorescent staining. J.B performed immuno‐histological staining's. H.D performed cryo‐EM experiments. L.D. performed animal studies and plug assays. M.I.Z interpretation of data and contributed to writing the manuscript. K.B. and J.A.D and K.B performed and analysed SILAC mass‐spectrometry experiments. M.V Contributed to writing the manuscript. M.W. contributed to hypothesis generation and interpretation of the data. K.M.A.R coordinated the project, developed the hypothesis, interpretation of the data, and contributed to writing the manuscript.

## CONFLICTS OF INTEREST

None.

## Supporting information

Supporting Information

Supporting Information

Supporting Information

Supporting Information

Supporting Information

Supporting Information

Supporting Information

Supporting Information
