# Supplementary material for: Secretion of pro‐angiogenic extracellular vesicles during hypoxia is dependent on the autophagy‐related protein GABARAPL1
Source: J Extracell Vesicles. 2021 Dec 2;10(14):e12166. doi: 10.1002/jev2.12166 (PMC8640512; doi:10.1002/jev2.12166)
Supplement: Supplementary file 1 — Supporting Information [file JEV2-10-e12166-s001.pdf]

A

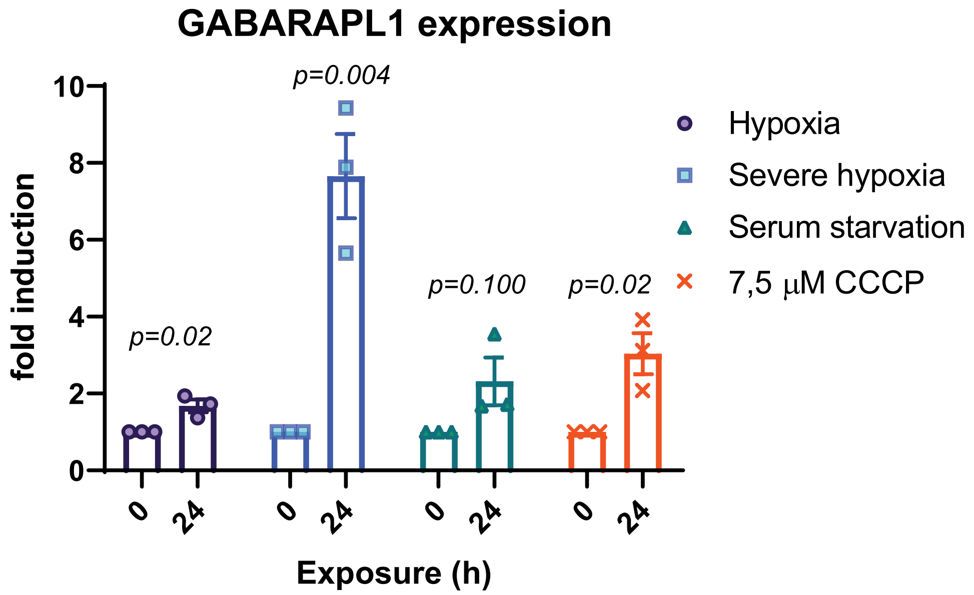

Supplementary Fig 1. (A) mRNA expression levels of GABARAPL1 exposed to different external stresses. (t-test unpaired, 2-tailed, mean ± SEM n=3)
