# Supplementary material for: Secretion of pro‐angiogenic extracellular vesicles during hypoxia is dependent on the autophagy‐related protein GABARAPL1
Source: J Extracell Vesicles. 2021 Dec 2;10(14):e12166. doi: 10.1002/jev2.12166 (PMC8640512; doi:10.1002/jev2.12166)
Supplement: Supplementary file 3 — Supporting Information [file JEV2-10-e12166-s008.pdf]

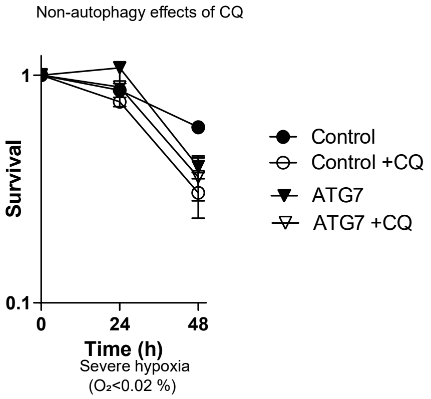

Supplementary Fig 3 Survival studies show no additional effect of CQ on survival of ATG7 deficient cells. CQ=chloroquine
