# Supplementary material for: Secretion of pro‐angiogenic extracellular vesicles during hypoxia is dependent on the autophagy‐related protein GABARAPL1
Source: J Extracell Vesicles. 2021 Dec 2;10(14):e12166. doi: 10.1002/jev2.12166 (PMC8640512; doi:10.1002/jev2.12166)
Supplement: Supplementary file 4 — Supporting Information [file JEV2-10-e12166-s002.pdf]

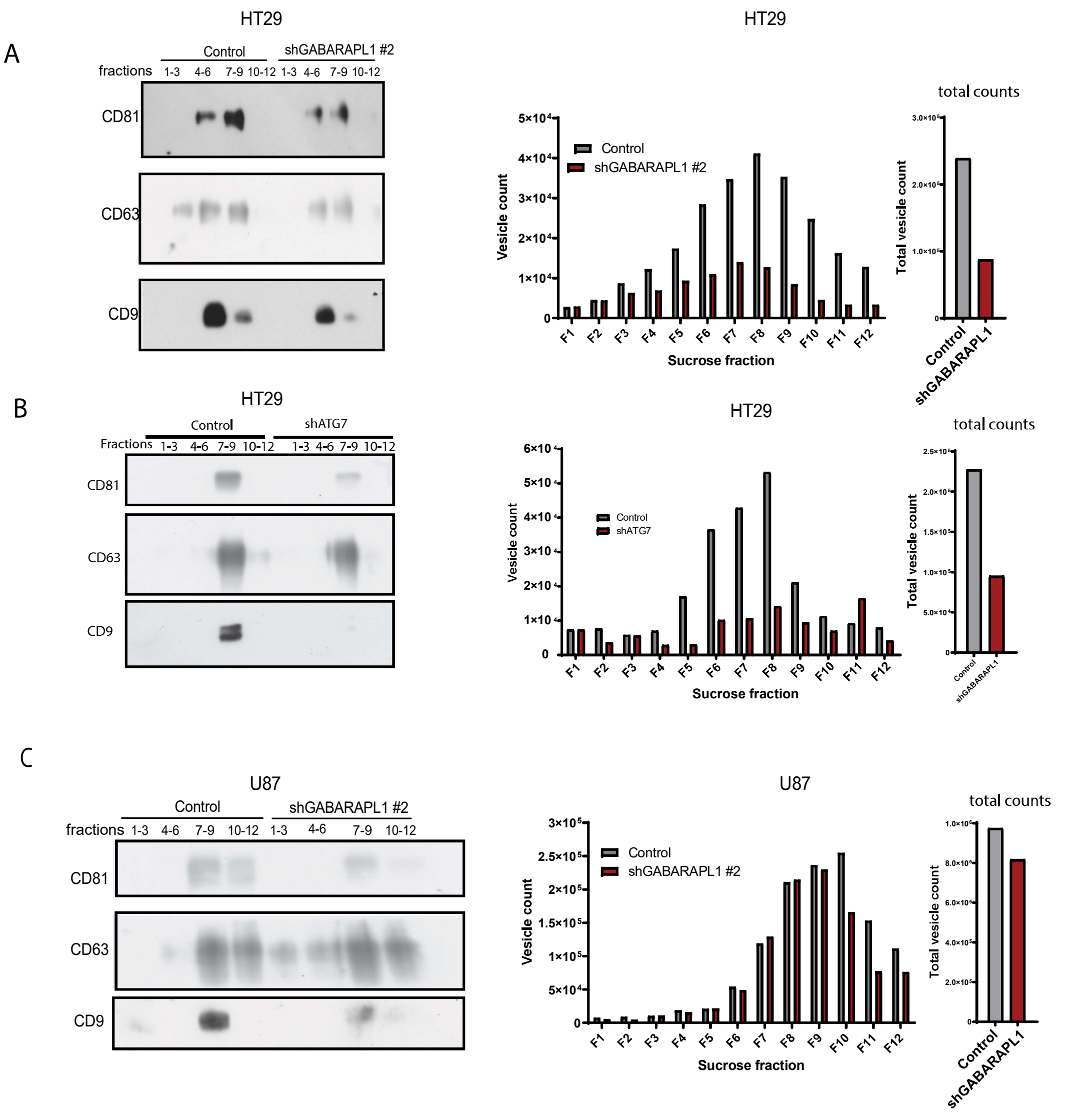

Supplementary Fig 4. A) Western blots of secreted EV show decreased marker expression. EVs are secreted by HT29 cells expressing an independent shRNA targeting GABARAPL1. Right panel shows decreased EV counts as measured by high resolution flow cytometry. B) Knockdown of ATG7 in HT29 cells shows a decrease in marker expression of secreted EVs isolated by sucrose density floatation. Right panel shows a decrease of EV counts as measured by high-resolution flow cytometry. C) Western blots of secreted EV show decreased marker expression. EVs are secreted by U87 cells expressing an independent shRNA targeting GABARAPL1. Right panel shows no changes in EV counts as measured by high resolution flow cytometry.
