# Supplementary material for: Secretion of pro‐angiogenic extracellular vesicles during hypoxia is dependent on the autophagy‐related protein GABARAPL1
Source: J Extracell Vesicles. 2021 Dec 2;10(14):e12166. doi: 10.1002/jev2.12166 (PMC8640512; doi:10.1002/jev2.12166)
Supplement: Supplementary file 5 — Supporting Information [file JEV2-10-e12166-s003.pdf]

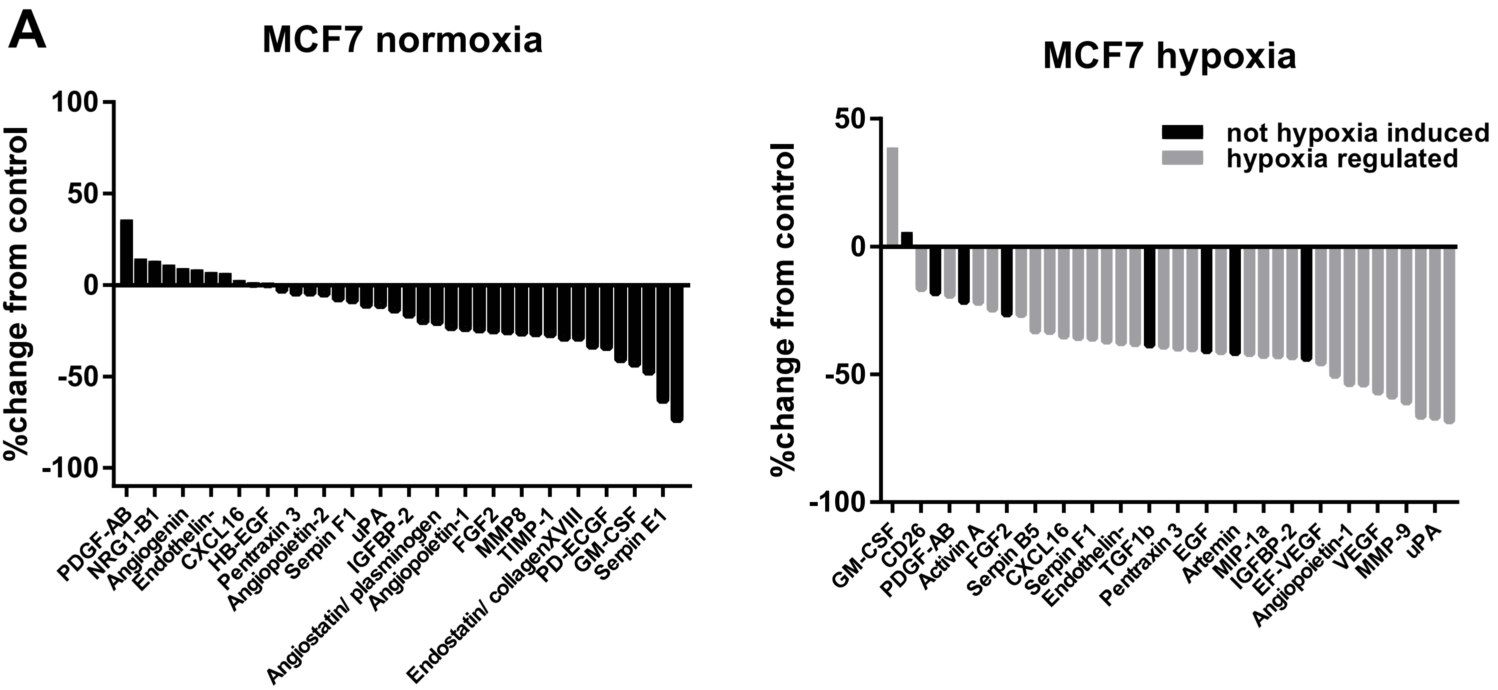

Supplementary Fig 5 (A) quantification of antibody arrays of MCF7 control and GABARAPL1 knockdown cells during ambient conditions
