# Supplementary material for: Secretion of pro‐angiogenic extracellular vesicles during hypoxia is dependent on the autophagy‐related protein GABARAPL1
Source: J Extracell Vesicles. 2021 Dec 2;10(14):e12166. doi: 10.1002/jev2.12166 (PMC8640512; doi:10.1002/jev2.12166)
Supplement: Supplementary file 6 — Supporting Information [file JEV2-10-e12166-s006.pdf]

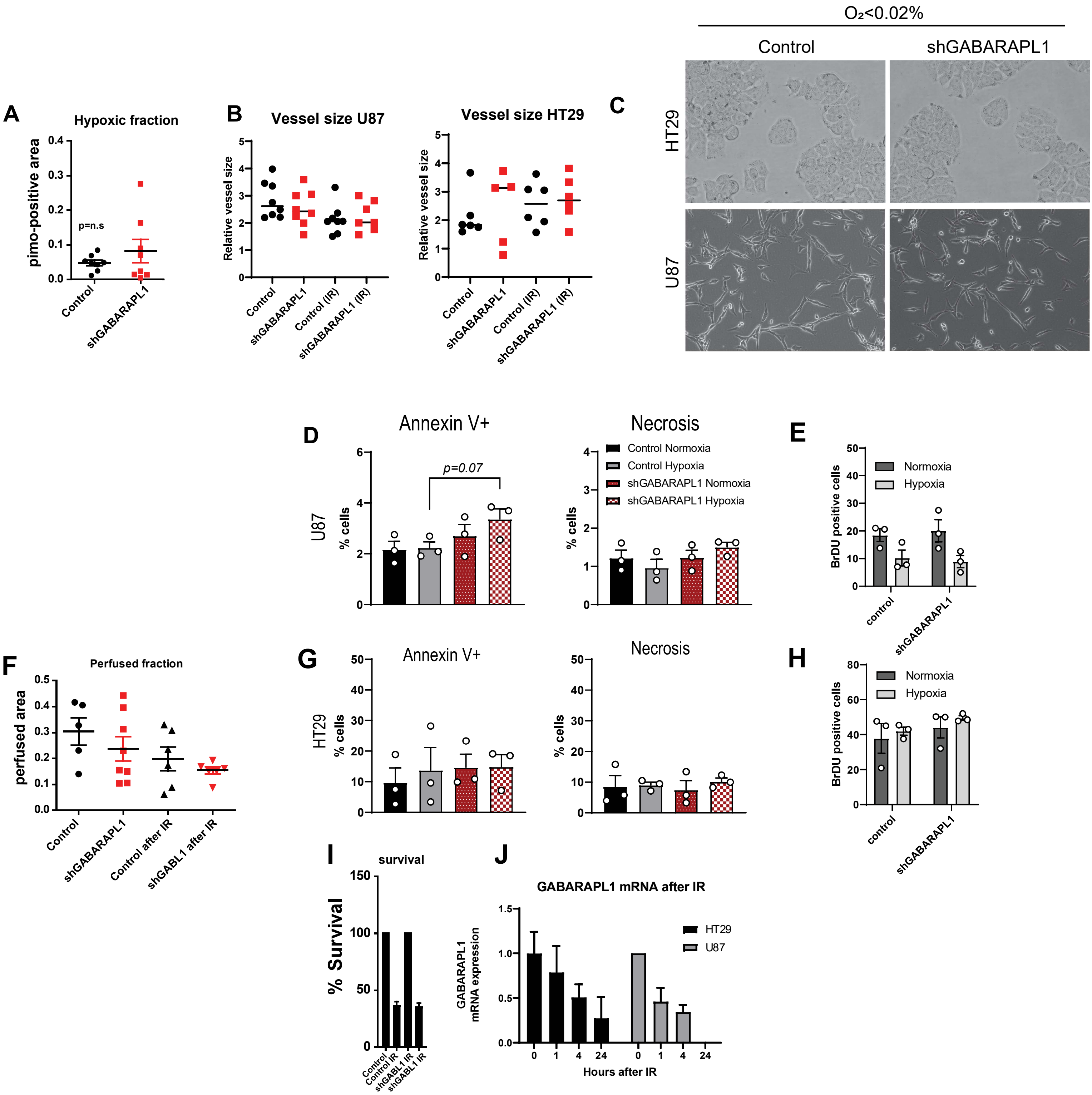

Supplementary Fig 6 (A) Hypoxic fraction of U87 xenografts as determined by pimonidazole immunohistochemistry. (B) Vessel size of U87 and HT29 xenografts of untreated and regrown tumors after irradiation (IR). Vessels were determined by 9F1/CD31 immunohistochemistry. Vessel size was calculated by Relative vessel area/Vessel density (RVA/VD). (C) Morphology of control and GABARAPL1 knockdown cells exposed to severe hypoxia. (D) Expression of Annexin V of U87 control and GABARAPL1 knockdown cells exposed to normoxia and severe hypoxia (24h) as determined by FACS analysis shows no that GABARAPL1 knockdown cells are more apoptotic during severe hypoxia. (n=3, t-test unpaired, 2-tailed, mean ± SEM). (E) Quantification of BrDU incorporation reveals no differences in proliferative capacity of U87 GABARAPL1 knockdown cells). (F) Vessel perfusion of U87 xenografts. (G) Expression of Annexin V of HT29 control and GABARAPL1 knockdown cells exposed to normoxia and severe hypoxia (24h) as determined by FACS analysis. (n=3, t-test unpaired, 2-tailed, mean ± SEM). (H) Quantification of BrdU incorporation reveals no differences in proliferative capacity of HT29 GABARAPL1 knockdown cells. (I) Clonogenic survival of HT29 control and GABARAPL1 knockdown cells after irradiation (single dose 6Gy). (J) GABARAPL1 mRNA expression is downregulated 24 after irradiation (single dose 6Gy) as determined by qPCR.
