# Supplementary material for: Secretion of pro‐angiogenic extracellular vesicles during hypoxia is dependent on the autophagy‐related protein GABARAPL1
Source: J Extracell Vesicles. 2021 Dec 2;10(14):e12166. doi: 10.1002/jev2.12166 (PMC8640512; doi:10.1002/jev2.12166)
Supplement: Supplementary file 7 — Supporting Information [file JEV2-10-e12166-s004.pdf]

Table 1

Gene Symbol	Description	Entrez GeneID	CONTROL / ShGABARAPL1	EV associated (exocarta. org)
ACTB	actin, beta	60	-27%	Yes
AMIGO2	adhesion molecule with Ig-like domain 2	347902	-35%	Yes
APLP2	amyloid beta (A4) precursor-like protein 2	334	-49%	Yes
ATP6AP1	ATPase, H+ transporting, lysosomal accessory protein 1	537	-29%	Yes
CD81	CD81 molecule	975	-20%	Yes
CLU	clusterin	1191	-23%	Yes
COL1A1	collagen, type I, alpha 1	1277	-43%	Yes
CTSB	cathepsin B	1508	-11%	Yes
CTSC	cathepsin C	1075	-25%	Yes
EPCAM	epithelial cell adhesion molecule	4072	-15%	Yes
FAT1	FAT atypical cadherin 1	2195	-29%	Yes
FRAS1	Fraser extracellular matrix complex subunit 1	80144	-24%	-
GCNT3	glucosaminyl (N-acetyl) transferase 3, mucin type	9245	-23%	Yes
GDF15	growth differentiation factor 15	9518	-37%	Yes
GLA	galactosidase, alpha	2717	-12%	Yes
GOLM1	golgi membrane protein 1	51280	-10%	Yes
GPC1	glypican 1	2817	-42%	Yes
GSN	gelsolin	2934	-34%	Yes
HEXA	hexosaminidase A (alpha polypeptide)	3073	-10%	Yes
HIST2H2BF	histone cluster 2, H2bf	440689	-47%	Yes
HSP90AB1	heat shock protein 90kDa alpha (cytosolic), class B member 1	3326	-19%	Yes
IGFBP4	insulin-like growth factor binding protein 4	3487	-20%	Yes
IGFBP6	insulin-like growth factor binding protein 6	3489	-18%	Yes
JAG1	jagged 1	182	-17%	Yes
LAMA5	laminin, alpha 5	3911	-29%	Yes
LAMC1	laminin, gamma 1 (formerly LAMB2)	3915	-26%	Yes
LGMN	legumain	5641	-13%	-
LOXL4	lysyl oxidase-like 4	84171	-13%	Yes
MET	MET proto-oncogene, receptor tyrosine kinase	4233	-24%	Yes
NEO1	neogenin 1	4756	-20%	Yes
NUCB1	nucleobindin 1	4924	-11%	Yes
PLOD2	procollagen-lysine, 2-oxoglutarate 5-dioxygenase 2	5352	-13%	Yes
PTPRF	protein tyrosine phosphatase, receptor type, F	5792	-51%	Yes
S100A11	S100 calcium binding protein A11	6282	-14%	Yes
SDF4	stromal cell derived factor 4	51150	-29%	Yes
SEMA7A	semaphorin 7A, GPI membrane anchor (John Milton Hagen blood group)	8482	-38%	-
SERPINE2	serpin peptidase inhibitor, clade E (nexin, plasminogen activator inhibitor type 1), member 2	5270	-53%	Yes
SPINK1	serine peptidase inhibitor, Kazal type 1	6690	-20%	Yes
TFF1	trefoil factor 1	7031	-22%	-
THBS1	thrombospondin 1	7057	-29%	Yes
TINAGL1	tubulointerstitial nephritis antigen-like 1	64129	-16%	Yes
TSPAN1	tetraspanin 1	10103	-28%	Yes
TUBB4B	tubulin, beta 4B class IVb	10383	-36%	Yes
TWSG1	twisted gastrulation BMP signaling modulator 1	57045	-33%	-
